# A scoping review of WeChat to facilitate professional healthcare education in Mainland China

**DOI:** 10.1080/10872981.2020.1782594

**Published:** 2020-06-23

**Authors:** Hui Luan, Miao Wang, Rebeccah L. Sokol, Shiyou Wu, Bryan G. Victor, Brian E. Perron

**Affiliations:** a Department of Social Work, School of Social Development, Tianjin University of Technology, Tianjin, China; b School of Social Work, University of Michigan, Ann Arbor, MI, USA; c School of Social Work, Arizona State University, Phoenix, AZ, USA; d School of Social Work, Indiana University, Indianapolis, IN, USA

**Keywords:** WeChat, professional education, healthcare, scoping review, mainland China

## Abstract

**Aim:**

WeChat is the most popular social media platform in mainland China, with over 1 billion active users. Although social media is widely used in professional healthcare education in western countries, research on WeChat-based education in healthcare in mainland China is disparate and not systematic. The current study seeks to address this gap.

**Method:**

A scoping review was conducted to systematically describe studies of WeChat use in professional healthcare education. A comprehensive search involving three international databases in English and Chinese literature was conducted in April 2019. Articles were retained in this study if they were original studies that used WeChat as a tool to facilitate healthcare education in mainland China.

**Results:**

25 studies met the inclusion criteria and the majority of studies were either experimental or quasi-experimental. WeChat was used in both university settings and hospital settings. Hybrid education–which integrates WeChat education and face-to-face education–was more common in university settings, whereas hospitals used a combination of hybrid and WeChat-only strategies. Significant heterogeneity was observed regarding the type of accounts and methods for delivering content and facilitating online conversations. A majority of studies found positive outcomes with WeChat education.

**Conclusions:**

This scoping review addressed a large gap in knowledge about the usage of WeChat in professional healthcare education. Of the existing studies identified, we observed considerable promise for future practice. We provide additional suggestions for conducting future research involving patients and other helping professionals in healthcare education to expand the usage of WeChat.

## Introduction

Social media has developed rapidly during the past decade and woven itself into nearly all aspects of daily life, with the most commonly used applications being Twitter, Facebook, and Instagram [1]. Social media has also emerged as new channels and tools for professional settings, especially within the health services [2–4]. Recent evidence demonstrates that social media is being used to facilitate health management [5,6,7], increase physician-patient communication [8,9], and deliver direct services [10,11].

In addition to facilitating and delivering health services, social media plays an important role in health professional education. Given its capabilities to disseminate information efficiently and facilitate communication outside of the traditional classroom, social media is widely recognized as an important educational tool [4,12–14]. Prior studies have found social media promotes students’ course attendance, learning engagement, interprofessional education, and professional development across many different disciplines [15,16].

In mainland China, many popularly-used social media apps (e.g., Twitter, Facebook, Instagram) are not accessible due to governmental policies. However, in 2011, a social media platform called WeChat was launched in mainland China and gained rapid and enormous popularity among Chinese communities all over the world. Currently, the estimated number of WeChat active users globally is over one billion [17]. WeChat functions in a manner similar to other social media platforms. Specifically, WeChat offers a free instant messaging application for smartphones, tablets, and laptops that operates similar to Facebook and other social media applications, with the primary functions of enabling communication and sharing of various types of media within a social network [18].

Given the similarities with other social media platforms, WeChat has the potential to be a tool to facilitate education across a wide range of disciplines. Given recent efforts of the Chinese government to innovate training for health professionals to achieve a variety of public health goals [e.g., Healthy China 2030 Program Outline; 19], WeChat may be an important educational tool for a range of health professionals in mainland China such as physicians, nurses, social workers, and psychologists. Despite the widespread use of WeChat in mainland China, the research on WeChat-based educational initiatives in mainland China is non-systematized and disparate. To date, we have no summary of the existing knowledge about the role of social media in professional healthcare education in mainland China.

The current study seeks to address this gap using a scoping review methodology, which involves describing the volume, nature, and characteristics of primary research [20]. The specific aims of this research are to describe: a) the overall amount of research on WeChat usage in healthcare education in Mainland China; b) the characteristics of studies that utilize WeChat-based healthcare education in Mainland China; and c) WeChat educational strategies in professional healthcare in Mainland China.

## Methods

### Data sources and search strategy

In the current study, we conducted a comprehensive search of the research that examines the role of WeChat in professional healthcare education. We searched for studies in English and Chinese languages. The data sources and search strategies for each language were different, so we describe the procedures separately, below. Both searches were current through April 2019.

#### English language studies

PubMed was our data source for English-language articles. This is the most comprehensive academic database for health services and medical education. We searched all available fields using a single term *WeChat* and limited the search results to peer-reviewed articles. We then performed a secondary and iterative search of Google Scholar to identify studies that might have been missed by PubMed.

#### Chinese language studies

For studies in Chinese language, we searched the China National Knowledge Infrastructure database (CNKI) and China Science and Technology Journal Database (CSTJ). The Chinese system of journals do not follow the same peer-review process as English-language journals. Thus, we were unable to provide a targeted search to identify articles that are known to be peer-reviewed. We limited the search results to Chinese Social Science Citation Index journals (CSSCI), Chinese Science Citation Database journals (CSCD), and Key Journals Index journals (KJI). The journals and databases are recognized as the most authoritative collections of empirical research in China. Master’s and PhD thesis were excluded. Since WeChat studies are commonly conducted in China, we used the following query (translated from Chinese to English; see Appendix A for the search terms in Chinese) to identify Chinese articles in Chinese databases:

*WeChat* AND (*health* OR *care* OR *disease* OR *illness* OR *disabled* OR *injury* OR *psychological* OR *health behavior* OR *diagnosis* OR *treatment* OR *surgery* OR *medical* OR *clinical*)

This search strategy was applied to all the fields, including title, keywords, and abstract. We also note that terminology related to ‘education’ and ‘training’ was excluded to keep our search as broad as possible, given the number of synonyms in the Chinese language that relate to these concepts.

### Inclusion criteria

Our primary aim was to obtain empirical studies or structured literature reviews involving WeChat as part of an educational or training initiative among health professionals. Studies had to be conducted in mainland China. We specifically excluded the special administrative regions of China (i.e., Hong Kong, Macau, and Taiwan) given the differences in economic development and healthcare systems. Our definition of healthcare education was broadly defined to include training for different types of health professionals (e.g., physicians, nurses, physiotherapists, medical social workers, and psychologists).

### Review process

Our review was performed in a two-step process (Appendix B). We first reviewed the titles and abstracts of articles identified from our data search to identify studies that met our inclusion criteria. Two study authors who were fluent in Chinese and English conducted the initial screening. A reliability analysis showed perfect agreement in the decisions for inclusion and exclusion of articles. Following the initial screen, we then obtained and reviewed full-text articles to confirm inclusion.

Overall, 99 English-language articles were identified in PubMed for review, and no additional articles were identified using Google Scholar. Three of these studies met our final inclusion criteria. A total of 954 Chinese-language articles were identified in the initial review. After two-step reviewing, 22 were retained. Overall, this review yielded a total of 25 articles specifically related to professional healthcare education using WeChat in mainland China.

### Data extraction and coding

We developed a protocol for extracting and coding different characteristics of each study identified in the review. Specifically, we identified the study setting, focusing on whether the education was provided in university, hospital or other medical organizational settings. We also examined study characteristics (i.e., research design, sample size, study duration), education topics addressed within the study, the professional discipline of participants and instructors, and strategies for facilitating education using WeChat (i.e., education format, WeChat accounts utilized, and interactions in different accounts).

Regarding educational strategies, we coded the education format to indicate whether WeChat education was used exclusively without face-to-face education (*online-only*) or integrated with face-to-face education (*hybrid*). If the educational format was categorized as a hybrid, we furtherly identified at what stage WeChat education was integrated with face-to-face education, namely if WeChat was utilized *before, during*, and/or *after face-to-face* education.

We also coded the type of WeChat account that was used to facilitate the educational process. WeChat has two different types of accounts, *individual* and *public* accounts. An individual account is used similarly to individual Facebook and other social media accounts, whereby an individual can interact with her or his network by sending and receiving texts, images, voice messages, videos, documents, and instant video conferencing. Individual accounts also allow ‘groups’ to be created that allow communications with specific individuals in a network. Since interaction is one of the most important components of the learning experience [21], we also coded the structure of interactions within individual accounts, focusing on whether the interactions were among just participants (learners-learners) or among participants and the course instructors (learners-instructors).

Public WeChat accounts are akin to Facebook and other social media accounts that businesses and organizations use to connect with their network audiences. The primary feature of a public account is the dissemination of information from the public account administrator to the participants. With the public accounts, we coded for three types of interactions that can be facilitated through the use of specialized apps. The first is *learners-instructors* interactions that occur through interactive commenting on posts made by the public account administrator. These interactions are among participants and with the instructor. The second type is *learners-learners* interactions, whereby learners interact with each other through the commenting feature, but no interaction with the instructors. The third type is *passive interaction*. This means that learners only receive information but there are no communications between participants or instructors.

## Results

A total of 25 studies were retained for inclusion in the current study. The majority of studies were in Chinese (*n* = 22) and spanned a period of six years (2014–2019). We summarized the study characteristics and WeChat educational strategies based on their study settings respectively.

### University settings

#### Study characteristics

As summarized in Table 1, 11 studies were conducted in university settings. Among these, 10 were published in Chinese. Six studies focused on teaching different types of skills (e.g., nursing skills, operational skills, emergency rescue skills, and medical laboratory skills) among undergraduates and nurses in the military medical universities. The other five studies focused on various topics to medical students in different majors (i.e., basic pathology, biochemistry and cellular biology, specific nursing) in general universities. The number of participants varied widely, ranging from 10 to 281 (mean = 119.7, median = 94). The intervention duration ranged from 2 months to 5 months (mean = 3.3, median = 3) based on a subset of studies that provided such information (*n* = 4). Among the 11 studies in university settings, only one study used online-only education with only WeChat [22]. The other ten studies used hybrid education which integrated WeChat education with face-to-face education.

**Table 1.** Study characteristics of WeChat-based educational interventions in university settings (n = 11).

Author	Education topic and learners	Research design	Sample size	Duration [Month)	Education format
Tian et al., [22]	Clinical thoracic surgery and skill; Medical students (undergraduates and postgraduates)	RCT	Intervention: 37; Control: 37	N/R	Online-only
Gao et al., [23]	Clinical pathology and laboratory skills; Medical students	RCT	Intervention: 33; Control: 33	N/R	Hybrid
Hua Zhang et al., [30]	Pediatric nursing; Medical students (undergraduates)	RCT	Intervention: 60; Control: 61	N/R	Hybrid
Lan et al., [25]	Emergency care; Medical students (undergraduates)	RCT	Intervention: 39; Control: 37	2	Hybrid
J. Zhong et al., [32]	Nursing and traditional Chinese medicine education; Medical students	RCT	Intervention: 61; Control: 62	4	Hybrid
Zheng et al., [31]	Basic pathology; Medical students (undergraduates)	RCT	Intervention: 57; Control: 57	N/R	Hybrid
Xiao et al., [24]	Experimental microbiology and medical laboratory skills; Medical students	Quasi- experimental	Intervention: 31; Control: 32	N/R	Hybrid
Guo et al., [29]	Internal medical nursing; Medical students (undergraduates)	Quasi- experimental	Intervention: 136; Control: 133	5	Hybrid
J. Wang et al., [28]*	Biochemistry and cellular biology; Medical students	Cross-sectional	N/R	N/R	Hybrid
Shi et al., [26]	Pre-job training of nursing and emergency rescue skills; Nurses in army hospitals	Cross-sectional	Intervention: 10	2	Hybrid
Sun et al., [27]	Nursing skills; Medical students	Cross-sectional	Intervention: 281	N/R	Hybrid

*Note*. Asterisks (*) indicated that the study was published in English language; N/R = Not reported

#### WeChat educational strategies

We present the strategies of WeChat education in university settings by their *education format, interaction type*, and *education results* (Appendix C). Among the studies in university settings, we observed only one online-only program [22]. In this study, WeChat was used to facilitate the application of *self-learning online* into university education, in which a protocoled learning plan was used to organize online education.

Modules aimed at facilitating online learning and teaching activities were established in a public account, and interfaces for learners and instructors were included. Public accounts with a learner interface supported online learners to access the course introduction, record learning notes, conduct a self-learning evaluation and course evaluation, and submit assignments. Public accounts with an instructor interface facilitated teaching with a primary focus on assessing students’ performance with respect to grading. Interactions among learners was promoted in WeChat, where personal learning doubts could be solved through group discussion among learners in a public account. This use of WeChat in online education promoted students’ self-learning ability, critical thinking ability, creative ability, and communication ability, as well as improved student test scores compared to students in traditional course learning [22]. No clear evidence was found to suggest that WeChat-based online education simulated traditional face-to-face education, since no significant differences in operational skills were found [22].

Ten studies used a hybrid format, integrating both face-to-face and WeChat education. Consistent with prior studies, the usage of the WeChat platform was supportive in expanding the course time and space among participants outside the classroom, in which WeChat was implemented in different stages of face-to-face education and served to multidirectional interaction, including learner-instructor and learner-learner interaction [23–32].

In the hybrid format studies, WeChat amplified course time by providing a convenient way of information sharing before class (*n* = 10). Among these studies, two implemented WeChat before the class education [23,30], and various online activities were also supported by WeChat platform. For instance, in Hua Zhang et al. [30]’s study, both individual and public accounts were used to facilitate learning activities before class. Online discussions among learners were guided by instructors using individual accounts, while delivery of course information and interactive commenting between learners and instructors were conducted via a public account.

WeChat also supported learning activities during class (*n* = 3). Specifically, teachers shared practice videos via individual accounts [25,32], or sent group discussion topics via a public account [26]. In addition, instant chatting via individual account allowed instructors to assess challenges and provide direct feedback to learners during class [25,32]. WeChat groups established in individual accounts also allowed students to upload practice assignments and to conduct peer assessments [25]. Thus, learner-learner interactions and learner-instructor interactions were promoted through WeChat during the course.

Moreover, WeChat permitted learners and instructors to conduct question-and-answer activities and share information after an in-person class or the conclusion of the course, thereby extending the pedagogical interaction beyond the classroom (*n* = 8). For instance, in [28]’s study, individual accounts were implemented to support continuing discussion among learners focused on case analysis, and to facilitate question-and-answer activities between learners and instructors after class. In other studies, course evaluation, simulation exercises, online tests, extensive learning resources, and preview course materials were provided after class via a public account, again extending pedagogical interaction (e.g., 29; 31).

Studies observed the outcomes and perceptions of hybrid education with the usage of WeChat. Overall, most of the studies indicated positive outcomes in a variety of indicators such as test scores, cognitive and communication abilities, course evaluation, and course satisfaction. However, a few studies found no significant associations between WeChat education and outcomes such as test scores, operational skills and course evaluation [23,29].

### Hospital settings

#### Study characteristics

As summarized in Table 2, 14 studies were conducted in hospital settings. Among these, 12 were published in Chinese language journals. Nine studies focused on pre-job and in-job training among nurses, and the other studies focused on clinical skills education among medical and nursing interns (*n* = 4; e.g., anesthesiology, nosocomial infection, and tracheotomy education) and the assistants in the operation room (*n* = 1; i.e., hand hygiene education). The number of participants varied widely, ranging from 39 to 585 (mean = 144.5, median = 102). The overall duration ranged from 1 month to 27 months (mean = 11.0, median = 8) based on the information provided. Of the 14 reviewed studies, six studies used online-only education with WeChat, and other eight studies used hybrid education which integrated WeChat and face-to-face education.

**Table 2.** Study characteristics of WeChat-based educational interventions in hospital settings (n = 14).

Author	Education topic and learners	Research design	Sample size	Duration (Month)	Education format
X. Wang et al., [33]	Pre-job training; Nurses	RCT	Intervention: 112; Control: 112	24	Online-only
Li et al., [45]	Intern training of prevention of nosocomial infections; Nurses	RCT	Intervention: 51; Control: 51	27	Hybrid
Haopeng Zhang et al., [44]	Clinical practice of anesthesiology; Medical students (Undergraduates)	RCT	Intervention: 21; Control: 20	8	Hybrid
Zhang et al., [43]	Clinical medicine and practice; Medical students (Undergraduates)	RCT	Intervention: 327; Control: 312	N/R	Hybrid
F. Wang et al., [46]*	Dementia-specific training; Nurses	RCT	Intervention: 36; Control: 36	3	Hybrid
Zhang et al., [34]	Wound care training; Nurses	Quasi-experimental	Intervention: 383	3	Online-only
Y. Zhong et al., [35]	Hand hygiene education of nosocomial infection prevention; Assistants in operation room	Quasi-experimental	Intervention: 301; Control: 284	16	Online-only
Chen et al., [36]	Education of elderly care; Nurses	Quasi-experimental	Intervention: 96; Control: 80	8	Online-only
Liu et al., [37]*	Continuing education in emergency department; Nurses	Quasi-experimental	Intervention:124	24	Online-only
Tian et al., [42]	Clinical education of in tracheotomy and dress technology; Medical students [Undergraduates)	Quasi-experimental	Intervention: 32; Control: 30	1	Hybrid
Yin et al., [40]	Pre-job training in operation room; Nurses	Quasi-experimental	Intervention: 39	3	Hybrid
Wang, [39]	Clinical practice of nursing; Medical students [Undergraduates]	Quasi-experimental	Intervention: 41; Control: 42	10	Hybrid
Zhang et al., [38]	Continuing education; Nurses	Cross-sectional	Intervention: 165	14	Online-only
Ouyang et al., [41]	Education of knowledge of HIV/AIDS; Nurses	Cross-sectional	Intervention: 295	1.5	Hybrid

*Note*. Asterisks [*] indicated that the study was published in English language. N/R = Not reported

#### WeChat educational strategies

The strategies of WeChat education in hospital settings were summarized by their *education format, interaction type*, and *education results* (Appendix D). Among the studies in hospital settings, less than half used online-only education (*n* = 6) and all of which utilized WeChat public accounts to conduct online learning activities. WeChat public accounts were recognized as useful for information delivery, instant feedback between learners and instructors [33,34,35], and facilitation of peer discussion among learners [36–38]. For instance, in X. Wang et al. [33]’s study, modules in a public account facilitated online training, career planning, academic communication, and mental health counseling for new nurses via public accounts. In addition, discussion modules via public accounts were found in [38]’s study, in which learners were allowed to post their opinions and conduct group discussion. In three studies, online examination was conducted after class via WeChat platform [34,36,37], allowing learners to arrange their time in a more flexible manner.

Among all the studies with online-only education format, increased test scores, better training performance with respect to implementation of clinical behaviors and training completion were found in WeChat education compared with traditional education. Meanwhile, two studies also reported positive satisfaction towards online education via WeChat platform [36,38].

Eight hospital-based studies used a hybrid format, integrating both face-to-face and WeChat education. The majority of studies in hospital settings evaluated clinical-based learning or practice-based learning combined with lecture-based education [except for 39, 40]. Therefore, WeChat was used as a supportive tool to deliver information, stimulate peer interactions and collect learning feedback throughout the education process [39–44], or to conduct online learning only after lecture-based education [45,46]. For instance, in one study a WeChat public account was used for information delivery before and after class without any interaction [44], allowing learners to preview information and promote self-learning without time restriction. In other examples, WeChat groups established via individual accounts engaged learners in group learning and promoted peer interaction before and after field training [41,43]. Organizing learning communities and stimulating instant feedback between learners and instructors via individual accounts were emphasized in practice-based training [42,43].

Two studies focused on the application of the *just-in-time*, which used WeChat to promote online engagement and self-guided learning [39,40]. Specifically, WeChat was used to help facilitate learning activities and delivery of course content. Moreover, WeChat was also used to help provide learners with feedback before class and promote interactions among learners and instructors after class. In addition, WeChat provided instructors with feedback information to make adjustments in course content.

WeChat was also used after lecture-based education with the purpose of knowledge reinforcement and interaction stimulation [45,46]. For instance, in F. Wang et al. [46]’s study, WeChat-based online learning activities were conducted after lecture-based education, aiming at reinforcing the education effect by providing timely feedback between learners and instructors as well as group discussion among learners.

Among the studies with hybrid education format, WeChat education integrated with lecture-based education showed significantly improvements in learning outcomes compared with non-WeChat education [46; 44]. Likewise, a number of studies involving practice-based WeChat education demonstrated improvement in not only test scores [43], but also clinical knowledge [e.g., HIV and dementia; 41, 46] and clinical behaviors [e.g., infection control, prevention behavior of HIV and emergency nursing; 45, 41, 42] compared to non-WeChat education. Other studies showed WeChat-based education to be positively associated with learning ability [39] and course satisfaction [40].

## Discussion

In this summary, we provide commentary on our findings related to learners and learner interactions, study design, and outcomes. We then consider how the findings from the current scoping review can suggest gaps and opportunities for innovating the delivery of healthcare education in Mainland China with social media. We also consider the strengths and limitations of our scoping review methodology and conclude with specific recommendations for advancing this area of research.

### Learners and learner-interactions

In the current study, we found a limited range of the different types of healthcare providers that were learners in the included studies. Specifically, the different types included medical students, nurses, and assistants in operation rooms. The limited range of learners can be explained by the current state of development of healthcare in Mainland China. Unlike Western countries such as the USA, there are a limited number of specialty healthcare providers in mainland China, the majority of healthcare systems in China do not even employ medical social workers. However, our current findings suggest that WeChat may be useful for promoting healthcare education across a variety of disciplines. Fields in Mainland China such as social work or psychology might therefore consider the integration of WeChat into future educational initiatives as they continue to develop.

The current study shows that learners were located in university or hospital settings within developed areas of China, such as Beijing and Shanghai. We did not find the use of WeChat to promote education in more remote and under-developed areas. Given the vast geography of China, the use of WeChat may be an important and untapped opportunity for increasing access to training, particularly in areas where professional health education is limited. [47],offered practical suggestions for enhancing education, communication and collaboration among health professionals in rural and remote areas, which can be a starting point to inform strategies that can be applied to China.

A hybrid format that used WeChat to promote discussions and information sharing as a supplement to face-to-face education was the most common for university initiatives. Only one study was completely online in university settings. Given the various university policies in China that determine the manner in which courses are organized and delivered [48], the short-term and medium-term opportunities for using WeChat are likely to remain in hybrid format. Research in a Western context has found that while learners often appreciate the increased flexibility that hybrid formats provide, there are also challenges related to reducing in-person interactions between learners and instructors [49]. Identifying whether and how the benefits and challenges of hybrid healthcare education manifest in Mainland China will be an important focus of subsequent empirical inquiry.

In hospital settings, online education delivered through WeChat was a commonly used strategy for continuing education, pre-job and on-the-job training. The main purpose of professional education in hospital settings was not only to learn systematic knowledge in their major areas, but to master job-related knowledge and skills that could be applied directly in their clinical work. Because healthcare professionals in hospitals have limited opportunities to attend full-semester courses in classroom settings, the use of WeChat to facilitate education appears especially promising for healthcare professionals. The policies for continuing education within the hospital settings are also much less formalized than the university setting. Thus, healthcare educators and researchers may have further opportunities to innovate with different formats and the specific use of WeChat for communication and collaboration.

Considerable heterogeneity was observed in the different types of WeChat accounts that were used. The usage of WeChat accounts and the modules activated in WeChat accounts diversified the usage of WeChat education between settings as well. Individual accounts were used commonly in university settings where interaction and instant communication was highlighted in modern university teaching [50], and this function was fully used in individual accounts [51]. Public accounts were commonly used in hospital settings, as they were beneficial for the hospitals. The well-running of WeChat public accounts could attract more subscribers from both regular users and their own working staff [52], thus promoting hospitals’ reputation and the exchange of knowledge from different departments and disciplines among a large audience size [37].

Despite the differences in account types, all accounts provided some capacity to promote interactions and information sharing among learners and instructors. We observed a mix of interaction strategies among the different studies, but the heterogeneity prevented systematic comparisons to suggest whether certain types of interactions were more effective than others. Thus, testing different account types will not be nearly as important for knowledge building as the specific communication strategies and educational content. We did not observe a clear role of theory that guided the course development and specific use of WeChat. Future studies may benefit from more actively integrating theory, which can lead to more rigorous hypothesis testing and research design.

### Study design, process, and outcomes

The heterogeneity of study design was a primary barrier to making systematic comparisons across studies. Approximately one-third of all the studies used a randomized controlled trial (RCT) design, which helped build confidence for establishing the specific mechanisms for observed educational outcomes. However, the majority of the RCTs did not contain enough descriptive information to assess the experimental quality. Although many of the studies included both a pre- and post-test, there were no longer-term follow-ups that were available to establish the level of knowledge retention and decay over time.

Course satisfaction was the most common educational process examined. Satisfaction scores were higher in all WeChat-based courses compared to traditional formats. Although we are not entirely clear on the specific mechanisms, the most likely explanation is due to the increased opportunities for interaction and support. Social media provides considerable opportunities for obtaining precise data on interactions, including the amount, timing, and directions. None of the studies obtained these kinds of data for assessing educational processes. This is an opportunity for advancing this line of research.

The major types of educational outcomes involved knowledge tests and skills tests. Similar to measures of process, we observed positive findings across the different educational outcomes. The positive findings can be interpreted in a couple ways. One possibility is that the use of social media has a general positive effect on traditional educational strategies in Mainland China. Given the widespread use of WeChat in China for personal communication, learners may find this as a convenient tool for accessing and engaging in education. Another possibility is that social media allows learners to receive additional education and instruction beyond the classroom to reinforce learning.

We think a more balanced view of the positive findings is necessary. Similar to the Western literature, there is a strong bias toward positive findings within the Chinese system of scholarship [53]. Thus, studies with non-significant findings may not have been published, or researchers have selectively reported only measures with positive findings. Ensuring that negative findings are publishable is a necessary step for advancing research broadly, especially the research on educational innovations.

### Study strengths and limitations

Our study makes important contributions toward organizing a highly disparate body of research on the use of social media to facilitate healthcare education in Mainland China. We consider the scoping review methodology an ideal approach to understand what research has been conducted, the major features of the research, and findings. Despite these strengths, our study results must be considered in the context of some notable limitations. The most notable problem is our inability to identify peer-reviewed research from the Chinese databases. More specifically, Chinese journals do not have the same editorial and review processes as Western journals. Thus, the studies may not have been subjected to a thorough peer-review process to help ensure quality. Moreover, as previously mentioned, we were unable to systematically review the research given the lack of information provided.

Finally, our study was limited to the use of WeChat. Although this is the most common form of social media in Mainland China, we want to recognize that we did not include other social media platforms that may be relevant to healthcare education, such as Weibo (similar to Twitter), YouKu (similar to YouTube), and Tik-Tok (short-form mobile videos). Future research should consider summarizing the use and effectiveness of these tools to build a comprehensive understanding of using social media to promote education in China.

### Next steps

Although the research on using WeChat to facilitate healthcare education is limited, we think the initial empirical base shows considerable promise for future development. Because of the significant role of educational policy in Mainland China, we think educators can promote the development of social media in healthcare education by identifying the free parameters or opportunities they have to innovate the curriculum. Embedding new strategies within a sound theoretical framework for learning and engagement is arguably one of the most practical steps for building a rigorous empirical base. We also think enhancing the research through a mixed-method design can help build a better understanding of the learner experience, offering insights into ways to strengthen effective processes and identifying new opportunities to innovate. Moreover, further studies are required to involve patients and other helping professionals in healthcare education to expand the usage of WeChat. By doing so, the role of social media within the Chinese context can be more effectively integrated into the training of healthcare professionals and responsive to a variety of public health goals put forward by the national government.

## Supplementary Material

Supplemental Material
