# Supplementary material for: A scoping review of WeChat to facilitate professional healthcare education in Mainland China
Source: Med Educ Online. 2020 Jun 23;25(1):1782594. doi: 10.1080/10872981.2020.1782594 (PMC7482650; doi:10.1080/10872981.2020.1782594)
Supplement: Supplemental Material [file ZMEO_A_1782594_SM4858.docx]

**Appendix A: Search strategy of keywords sets of the original Chinese query**

主题 = *微信* AND (*健康* OR*护理* OR *疾病* OR*病* OR *残疾* OR*伤害* OR *心理* OR *健康行为* OR *诊断* OR *治疗* OR *手术* OR *医疗* OR *临床*)

**Appendix B: Figure of workflow of article selection of WeChat usage in professional healthcare education in mainland China**


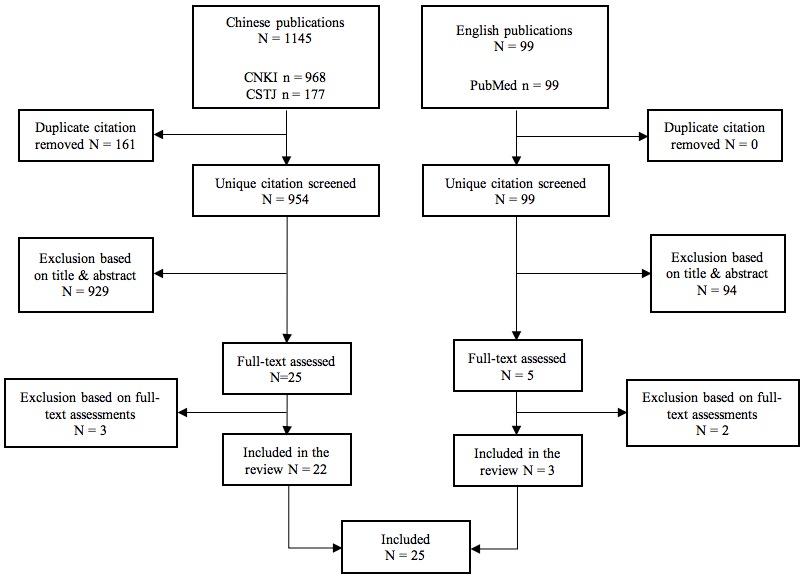


**Appendix C: Table of educational strategies across WeChat-based educational interventions in university settings (n = 11)**

| **Author** | **Education format** | | | **Interaction type** | | | **Education results** |
| --- | --- | --- | --- | --- | --- | --- | --- |
|  | Hybrid | | | Learners- Instructors | Learners-Learners | Passive |  |
|  | Before class | During class | After class |  |  |  |  |
| Tian et al., 2018** | - | - | - | - | P | - | Test scores, self-learning, critical thinking, creativity & communication ability ↑; No significant improvement in operations skills. |
| Gao et al., 2017 | I | - | - | I | I | - | No significant improvement in test scores, operational skills & course evaluation. |
| Hua Zhang et al., 2018 | I, P | - | **-** | I, P | I | - | Test scores, self-learning ability & critical thinking ability ↑ |
| Lan et al., 2018 | I | I | I | I | I | - | Test scores, self-learning ability & course evaluation ↑ |
| J. Zhong et al., 2017 | P | I | I | I | I | P | Test scores & operational skills ↑ |
| Shi et al., 2018 | P | P | I, P | I, P | - | - | Course satisfaction ↑ |
| Zheng et al., 2017 | I | - | I | I | I | - | Test scores ↑ |
| Xiao et al., 2018 | P | - | P | P | - | - | Test scores ↑ |
| J. Wang et al., 2017* | P | - | P | P | - | - | Test scores & course satisfaction ↑ |
| Sun et al., 2018 | P | - | P | - | - | P | Course satisfaction ↑ |
| Guo et al., 2017 | I | - | P | I | I | P | Test scores, self-learning mood & self-learning ability ↑ |

*Note*. I indicated *individual accounts*, P indicated *public accounts*. Dash (-) indicated *not applicable* or *not used*. ↑ indicated result was significantly improved. Asterisks (*) indicated that the study was published in English language. Double Asterisks (**) indicated that the study’s education format was online-only.

**Appendix D: Table of educational strategies across WeChat-based educational interventions in hospital settings (n = 14)**

| **Author** | **Education format** | | | | **Interaction type** | | | **Education results** |
| --- | --- | --- | --- | --- | --- | --- | --- | --- |
|  | Online- only | Hybrid | | | Learners- Instructors | Learners-Learners | Passive |  |
|  |  | Before class | During class | After class |  |  |  |  |
| X. Wang et al., 2017 | P | **-** | **-** | **-** | P | - | - | Test scores ↑ |
| Zhang et al., 2015 | P | - | **-** | - | - | P | - | Course satisfaction ↑ |
| Y. Zhong et al., 2017 | P | - | **-** | - | P | - | - | Implementation rate of hand washing ↑; Nosocomial infection rate & average hospitalization day of patients ↓ |
| Chen et al., 2018 | P | - | **-** | - | P | P | - | Test scores & course satisfaction ↑ |
| Liu et al., 2018* | P | - | **-** | - | - | P | - | Test scores & training completion rate ↑ |
| Zhang et al., 2014 | P | - | **-** | - | P | - | - | Test scores ↑ |
| Haopeng Zhang et al., 2018 | - | P | - | P | - | - | P | Test scores ↑ |
| Ouyang et al., 2016 | - | I | - | I | I | I | - | Knowledge & prevention behaviors of HIV ↑ |
| Zhang et al., 2019 | - | I | - | I | I | I | - | Test scores ↑ |
| Tian et al., 2016 | - | I | I | I | I | I | - | Test scores, pass rate of emergency nursing & clinical knowledge, accuracy rate of aseptic technique ↑ |
| Wang, 2018 | - | P | - | P | P | P | - | Test scores & self-learning ability ↑ |
| Yin et al., 2016 | - | P | - | P | P | - | - | Test scores & course evaluation ↑ |
| Li et al., 2018 | - | - | - | P | - | P | - | Test scores & implementation rate of infection control ↑ |
| F. Wang et al., 2017* | - | - | - | I | I | I | - | Dementia knowledge and attitudes & course satisfaction ↑ |

*Note*. I indicated *individual accounts*, P indicated *public accounts*. Dash (-) indicated *not applicable* or *not used*. ↑ indicated result was significantly improved. ↓ indicated result was significantly decreased. Asterisks (*) indicated that the study was published in English language.
